# Assessment of significance of features acquired from thyroid ultrasonograms in Hashimoto's disease

**DOI:** 10.1186/1475-925X-11-48

**Published:** 2012-08-16

**Authors:** Robert Koprowski, Witold Zieleźnik, Zygmunt Wróbel, Justyna Małyszek, Beata Stępień, Waldemar Wójcik

**Affiliations:** 1 Department of Computer Biomedical Systems University of Silesia, Institute of Computer Science, Będzińska 39 str., 41-200, Sosnowiec, Poland; 2 Internal Medicine Practice, Dworcowa 25/5 str., 41-902, Bytom, Poland; 3 Faculty of Electrical Engineering and Computer Science, Lublin University of Technology, Nadbystrzycka 38 D str., 20 – 618, Lublin, Poland

**Keywords:** Image processing, Hashimoto, Thyroid, Ultrasonograms

## Abstract

**Introduction:**

This paper concerns the analysis of the features obtained from thyroid ultrasound images in left and right transverse and longitudinal sections. In the image analysis, the thyroid lobe is treated as a texture for healthy subjects and patients with Hashimoto’s disease. The applied methods of analysis and image processing were profiled to obtain 10 features of the image. Then, their significance in the classification was shown.

**Material:**

In this study, the examined group consisted of 29 healthy subjects aged 18 to 60 and 65 patients with Hashimoto's disease. For each subject, four ultrasound images were taken. They were all in transverse and longitudinal sections of the right and left lobe of the thyroid, which gave 376 images in total.

**Method:**

10 different features obtained from each ultrasound image were suggested. The analyzed thyroid lobe was marked automatically or manually with a rectangular element.

**Results:**

The analysis of 10 features and the creation for each one of them their own decision tree configuration resulted in distinguishing 3 most significant features. The results of the quality of classification show accuracy above 94% for a non-trimmed decision tree.

## Introduction

### Basic definitions

Thyroid echogenicity is defined as the average brightness of pixels per unit area of the thyroid lobe and is expressed in decibels. The assessment of thyroid echogenicity is an important element of thyroid gland examination as it accompanies most changes in its histological structure. It may involve the entire gland or occur focally. The size of thyroid follicles [1] is the parameter which decides about the level of echogenicity. It goes down when there is a decrease in the amount of colloid in follicles and an increase in the number of cellular elements [2].

Thus, high echogenicity is determined by follicles large in diameter, which are found in parenchymatous goitre and nodules composed of follicles with a high content of colloid [1].

Echogenicity is low in places where follicles are small in diameter or they have been destroyed or there are inflammatory infiltrations or blood flow has increased [2].

### Qualitative methods for echogenicity assessment

There are several recognized ways of assessing thyroid echogenicity. In the 80's and 90's, it was assessed in relation to the tissues which surround the thyroid gland. Thyroid echogenicity was compared with a sternocleidomastoid muscle and subhyoid muscles. Echogenicity lower than that of these muscles was classified as reduced [1-5]. When echogenicity was higher, it was classified as normal. This method of assessment is widely used today.

Raber et al. [6] expanded the scale by introducing a three-stage system of thyroid echogenicity assessment. The system incorporated echogenicity of the salivary gland as an additional reference point [4].

### Quantitative methods for echogenicity assessment

At the end of the 90’s, first papers appeared in which a subjective assessment of echogenicity was avoided, and echogenicity was analyzed on the basis of the computed gray scale. It demonstrated practical utility of such an assessment in autoimmune thyroid diseases [1,7-11]. However, this method was not introduced to common clinical practice because the authors used various tools to measure image echogenicity. The image itself was obtained after image pre-processing, so instrument settings had a major impact on the obtained results. For this reason, the results were not consistent in these papers.

Irrespective of that, numerous studies have been carried out on the analysis of thyroid ultrasound images without profiling methods to Hashimoto’s disease. They were done by Mailloux et al. [8,12,13] and concerned the analysis of echogenicity as well as the histogram analysis in the analysis of thyroid lobes treated as a texture. Then, the approach was modified to texture analysis by introducing Haralick's coefficients to Co-occurrence matrices. It was done by Smutek et al. [14] and Bastanfard et al. [15]. There were also works on: fuzzifying the local binary patterns [16] and fuzzy grey-level histograms [17]. In papers [18,19], the authors used support vector machines in the analysis of thyroid lobes. In 2011, W. Zieleźnik et al. [12] showed the possibility of measuring thyroid echogenicity in decibels based on the program currently available in most ultrasound scanners which is used for the evaluation of atherosclerotic plaque and thrombus. The analysis shows that the measurement of echogenicity of the proximal part of the gland (from the front surface to the common carotid artery) is most reliable. The transducer frequency was set at 10 MHz and the option of harmonic imaging was disabled. In such settings, the cut-off point for Hashimoto’s disease is -69 dB [17]. The measurement was performed on source images. The measurement tool is widely available and unified.

## Examined group

In this paper, the examined group were:

 29 healthy subjects aged 18 to 60,

 65 patients with Hashimoto’s disease.

For each subject, four ultrasound images were made in transverse and longitudinal sections of the right and left lobe of the thyroid, which gave 376 images in total. This group was divided in equal proportions into learning, validation and test groups.

## Method

### Preliminary image analysis

*L*_*GRAY*_ input image in DICOM format, which is the source image recorded in GE ultrasound machine with a resolution of *M*× *N* = 614×816 pixels, is filtered using a median filter whose mask size is *M*_*h*_×*N*_*h*_ = 3×3. The filtered image *L*_*MED*_ provides the basis for further image analysis. In this image, derived from the learning, validation and test groups, an expert marks manually the area of analysis – it is usually the upper part of the thyroid lobe. The selected area is the only area marked manually. In this study, the area is rectangular and, in particular, square. Papers [20,21] and [22] describe a fully automatic way of marking an area of the thyroid. However, a clearly visible artery is the basis for its operation. In pathological cases the arterial lumen gets narrowed reducing the effectiveness of automatic operation of the algorithm. Thus, the algorithm described in papers [21] and [22] can be regarded as the operator support.

In the marked area *L*_*S*_ with a resolution of *M*_*s*_×*N*_*s*_, a morphological and statistical analysis is performed with the use of well-known techniques of analysis and image processing. These techniques were also suggested by the authors.

### Statistical and morphological analysis of the image

The statistical and morphological image analysis was shown based on the example of Hashimoto’s disease which is characterized by [23,24]:

 decreased echogenicity,

 sometimes heterogeneous structure,

 sometimes fibrosis seen as hyperechogenic linear structures.

On the basis of medical evidence concerning images of the thyroid lobes, two measured characteristic parameters for Hashimoto’s disease were formulated:

 an average brightness value after the removal of clear follicles of any diameter,

 measurement of statistical parameters of follicles whose size and shape is not strictly defined, mainly the number of their occurrences.

The measurement result of these parameters should not be sensitive to the size of the analyzed image *L*_*S*_ (normalization).

Such a general formulation of features is due to a very large individual variation and the dependence of results from the method of measurement. The texture analysis methods have been defined on the basis of these characteristics, taking into consideration the analysis of minimum values after the elimination of white follicles. A method for the analysis of clear textures, in general, objects, has also been suggested. Among the known methods for texture analysis [25] and on the basis of papers [12-19], the analysis of the input image *Ls* with statistical and morphological methods have been suggested, obtaining10 attributes defined as follows:

***w(1) – average image power spectrum defined as:***

(1) $$w\left(1\right)=\frac{\left(\sum_{M}^{p=1}\sum_{N}^{q=1}L_{f}\left(p,q\right)\right)}{\left(M*N\right)}$$

Where *L*_*f*_ is Fourier transform:

(2) $$L_{f}(p,q)={\left|\sum_{m=1}^{M}\sum_{n=1}^{N}L_{s}(m,n)e^{-j2\pi pm/M}e^{-j2\pi qn/N}\right|}^{2}$$

*p,q* - row and column of the matrix *L*_*f*_*,*

*m,n* -row and column of the matrix *Ls*.

**Figure 1:**
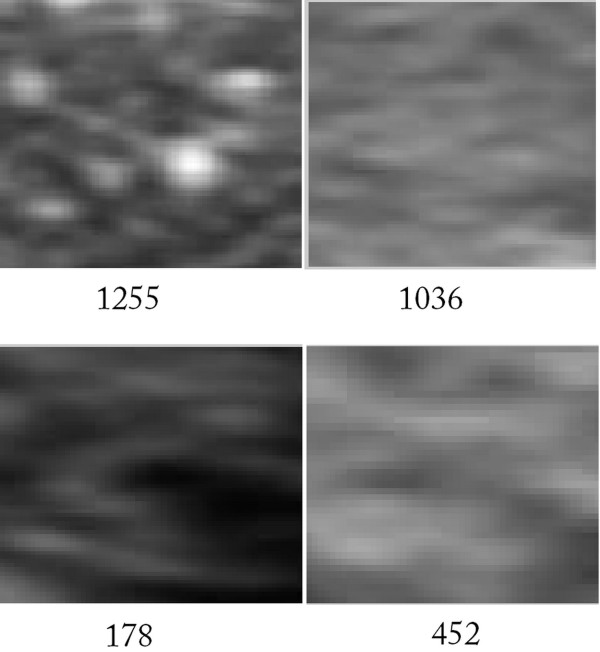
**Average image power spectrum*w*(1) for different pictures.** The values presented below images are values of the *w*(1) feature. A preliminary image analysis shows that the higher the homogeneity – lower frequency of changes, the lower the value of coefficient *w*(1). Higher, in terms of amplitude, changes, a higher frequency, a higher value of *w*(1). This feature is one of the 10 features that take part in creating a decision tree for classification of healthy subjects from patients. In this case, this feature is associated with Fourier decomposition and, to be more exact, with average image power spectrum.

Examples of results obtained for various textures of the thyroid lobes are presented in the figure below (Figure 1). The sample results obtained for various sample textures of the thyroid presented in Figure 1 show that the feature is a normalized sum of amplitudes occurring for individual harmonics. Diagnostically, the feature points to a systematic (with some assumed frequency) repetition of nodules, in general, artifacts on the thyroid lobe.

***w(2)- regional minimum value on the Ls image***

(3) $$w\left(2\right)=\min_{m\in \{1,..,M\},n\in \{1,..,N\}⋀Lo(m,n)0}\left(Ls\left(m,n\right)*Lo(m,n)\right)$$

where *L*_*O*_ – a binary image of local minima occurrence.

**Figure 2:**
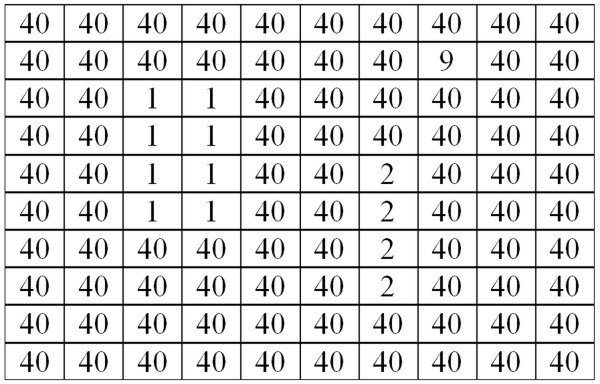
**Example of*L***_***S***_**input image.** An example of *L*_*S*_ image used later to demonstrate the next operation steps of algorithm calculating the value of coefficient *w*(2). The interpretation of brightness levels is not required here. In the image there are three isolated areas that contain the values 1, 2 and 9. They are an isolated focus. The surrounding pixel values equal to 40 are the backdrop. In the next step of the algorithm, those focuses (1, 2 and 9) are automatically separated from the backdrop (the values of 40). The number of pixels in the focus is not significant here. What matters are their surroundings - in this case the values of 40.

**Figure 3:**
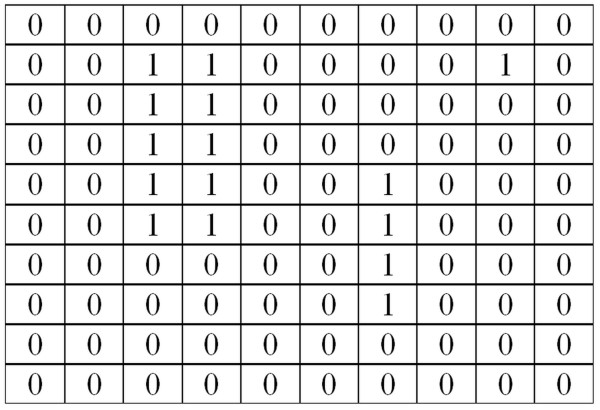
**Resultant*L***_***O***_**image with marked areas, within which the minimum values are calculated.** The methodology of computing the value of *w*(2) is clearly visible. Local minimum values are denoted as “1”. The pixel values "1" in the image *Lo* correspond to the marked focuses in the image *Ls* (values 1, 2 and 9). Thus the algorithm has found a local minimum and accordingly separated it from the backdrop (the backdrop has the values of 40). In this case, each minimum (the values of 1, 2 and 9) surrounded by the backdrop (by the values of 40) has been correctly separated. The focuses thus designated, marked with the values "1", are the basis for further analysis.

An operation of this function (3) is based on the calculation of the average of local minima. An example of the area *L*_*O*_, from which the values of local minima are calculated, is shown below (Figure 2 – an example of input image, Figure 3 –*L*_*O*_ image).

The minima indicated as values „1” in the image *Lo* (Figure 3) indicate the pixels involved in the calculation of the average value and, therefore, the value *w*(2). This feature provides reliable information on the average brightness of an image after the removal of clear artifacts and distortions, understood both as nodules as well as small inclusions or isolated bright pixels.

***w(3) – smoothness*** where

(4) $$w\left(3\right)=1-1/(1+w_{\mathrm{STD}}{}^{2})$$

(5) $$w_{\mathrm{STD}}=\sqrt{\frac{1}{M*N}\sum_{M}^{m=1}\sum_{N}^{n=1}\left(Ls\left(m,n\right)-\bar{Ls}\right)}$$

$\bar{Ls}$ the average brightness *Ls*.

Smoothness defined by the formula (4) is relatively easy to interpret because it is a standardized measure based on the standard deviation of the average. If STD and, therefore, the value *w*_*STD*_ increases, the value of the feature *w*(3) decreases. Thus, images of the thyroid, consistent in terms of changes in brightness of pixels, have the value of the feature *w*(3) close to zero. And vice versa, large changes in brightness and high values of STD result in an increase in the feature *w*(3) to the value of 1.

***w(4) – the minimum value of brightness on the L***_***S***_***image****, having removed all pixels, which number for the given brightness is smaller than 20% of the largest number of brightness pixels,* i.e. *:*

(6) $$hist\left(i\right)=\sum_{m=1}^{M}\sum_{n=1}^{N}k(i,m,n)$$

where

(7) $$k\left(i,m,n\right)=\{\begin{array}{l} 1\;if\;Ls\left(m,n\right)=1\\ 0\;other \end{array}$$

for *i* = 1,2,3,…,254,255.

(8) $$hist_{m}=\max_{i}\left(hist(i)\right)$$

where

(9) $$hist_{w}\left(i\right)=\{\begin{array}{l} hist\left(i\right)\;if\;hist\left(i\right)>0.2*hist_{m}\\ hist_{m}\;other \end{array}$$

(10) $$hist_{w}(i^{*})=\min_{i}\left(hist_{w}(i)\right)$$

The value of i* = w(4) calculated in this way is the minimum value in the analyzed area of the thyroid after the removal of distortions in the form of individual pixels with very low brightness. The threshold value of 0.2 was established arbitrarily on the grounds of noise present in the image *Ls* and preliminary analyses.

This feature is seemingly similar to *w*(2). However, in the case of a large number of bright nodules present in the analyzed area of the thyroid, the value of the feature *w*(4) remains constant while the value of the feature *w*(2) increases. The same situation occurs in the event of distortions in the form of pixels with low or zero brightness. In this case, they will be counted when determining the feature *w*(2). However, they will not be taken into account in the calculations of *w*(4).

*w(5) – position of the centre of GLCM (Gray-Level Co-occurrence Matrix) matrix gravity*

**Figure 4:**
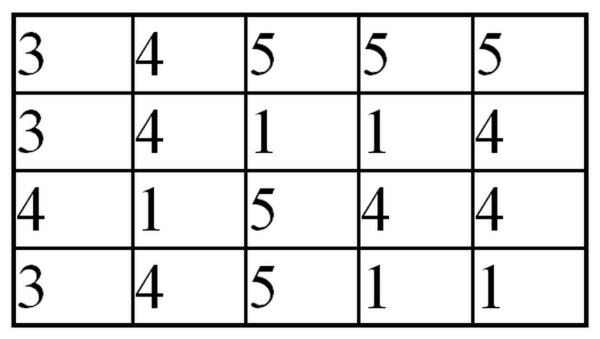
**Example of*L***_***S***_**matrix.** An example of *L*_*S*_ image used later to demonstrate the next operation steps of GLCM algorithm calculating the value of coefficient *w*(5). The interpretation of brightness levels is not required here. The next step will be the comparison of the neighborhood between horizontally neighbouring pixels. In this case there will be 16 neighborhoods. The value 16 results from four neighborhoods for each of 4 lines of the presented image (matrix). Among these 16 neighborhoods, there may appear both new neighborhoods as well as the previously detected ones.

**Figure 5:**
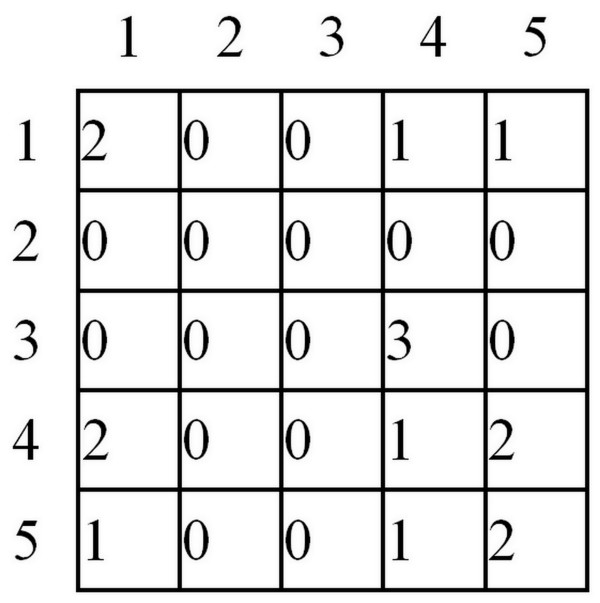
***L***_***GLCM***_**matrix created based on the*L***_***S***_**matrix.** The matrix presenting the total number of neighbourhoods between pixels. The number of neighbourhood is read selecting appropriate matrix column and row. For example, in row 3 and column 4 we read the value of 3. This means that on the image pixel 3 with the neighbouring pixel 4 occurred 3 times. All neighbourhoods (a total of 16) are calculated following this example. For this reason, the sum of the values in the matrix *L*_*GLCM*_ is 16. It should be noted that further transformations of the matrix *L*_*GLCM*_ are possible. They arise from the fact that the matrix *L*_*GLCM*_ is symmetric to the neighborhood - if the value 3 is adjacent to the value 4, then the value 4 is adjacent to the value 3. For further analysis, however, a distribution of neighborhoods and the number of their occurrences are vital. The further away form the main diagonal the neighborhood is detected and marked in the matrix *L*_*GLCM*_, the greater contrast it has. The neighborhoods in the lower right corner of the matrix *L*_*GLCM*_ refer to bright pixels whereas the ones in the upper left corner refer to dark pixels.

According to the definition, the matrix *L*_*GLCM*_ is determined on the basis of the number of neighborhoods of the closest pixels in the *x*-axis. Neighborhoods of pixels brightness are stored in rows and columns of the matrix *L*_*GLCM*_. For example, for the image in Figure 4, the neighborhood matrix can be written as shown in Figure 5.

For example, the neighbourhood 3 and 4 occurs three times, and therefore the value 3 is stored in the third row and fourth column of the matrix GLCM (*L*_*GLCM*_ -Figure 5). The other fields of the matrix GLCM are filled in the same way. After this stage, the matrix is summed with its transposition (if 3 neighbours with 4 then also 4 with the number of 3), and then normalized (to become independent from the resolution of the image *Ls*) and binarized to remove the effect of a small number of neighborhoods on the result, ie:

(11) $$L_{\mathrm{GLCMB}}(m,n)=\{\begin{array}{ccc} 1 & if & L_{\mathrm{GLCM}}(m,n)>p_{r}\\ 0 & if & L_{\mathrm{GLCM}}(m,n)\leq p_{r} \end{array}$$

where *p*_*r*_ – the threshold value defined as

(12) $$p_{r}=0.1*\max_{m,n}L_{\mathrm{GLCM}}(m,n)$$

For the *L*_*GLCMB*_ image obtained this way the centre of gravity of the object formed and its area are calculated, i.e.:

(13) $$x=\frac{\sum_{n}^{N}\sum_{m}^{M}L_{\mathrm{GLCMB}}\left(m,n\right)*m}{\sum_{n}^{N}\sum_{m}^{M}L_{\mathrm{GLCMB}}\left(m,n\right)}$$

(14) $$y=\frac{\sum_{n}^{N}\sum_{m}^{M}L_{\mathrm{GLCMB}}\left(m,n\right)*n}{\sum_{n}^{N}\sum_{m}^{M}L_{\mathrm{GLCMB}}(m,n)}$$

(15) $$w\left(5\right)=\sqrt{x^{2}+y^{2}}$$

(16) $$w\left(6\right)=\sum_{n}^{N}\sum_{m}^{M}L_{\mathrm{GLCMB}}(m,n)$$

Both the feature *w*(5) and *w*(6) determine the brightness and uniformity of brightness in the image. The feature *w*(5) provides information about the brightness of pixels and is not dependent on the contrast present in the analyzed texture of the thyroid. In the case of high contrast appearing in the image *Ls* as well as in the case of low contrast and homogeneous pixel values, the distance of the center of gravity from the origin of coordinates does not change, and so the value of the feature *w*(5). The range of contrast in different values of derivatives determines the value of the feature *w*(6). An increasing value of the feature *w*(6) means that in the image *Ls*, there are a lot of different combinations of pairs of pixels. The examples are the images with large changes in thyroid histological structure accompanied by various changes in brightness of adjacent pixels.

***w(7) to w(10) – the result of square-tree decomposition****– and exactly the percentage number of areas of size 1×1 – w(7), 2×2 – w(8), 4×4 – w(9), 8×8 – w(10) occurrences, obtained for a 10% threshold.*

**Figure 6:**
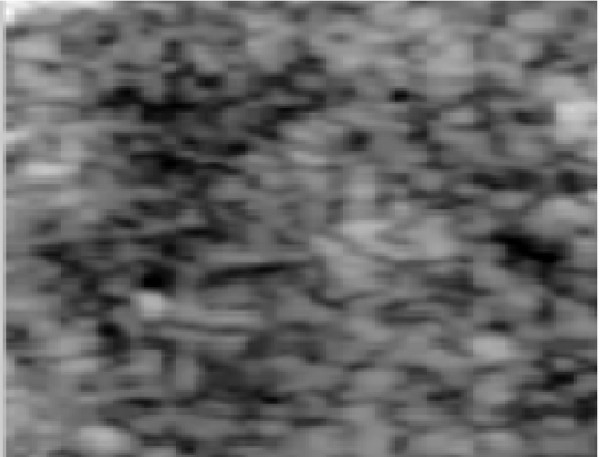
**Example of*L***_***S***_**image.** An example of *L*_*S*_ image used later to demonstrate the next operation steps of algorithm calculating the value of coefficients *w*(7) to *w*(10). In this example of the image, there are a number of bright objects well separated from the backdrop. The distribution of these objects is random, which makes their analysis with the use of classical methods difficult. In addition, the lack of uniformity in their size adds to the problem. The image *Ls* often does not contain well visible whole bright areas. Sometimes they are not visible at all or ragged at the edges of the image.

**Figure 7:**
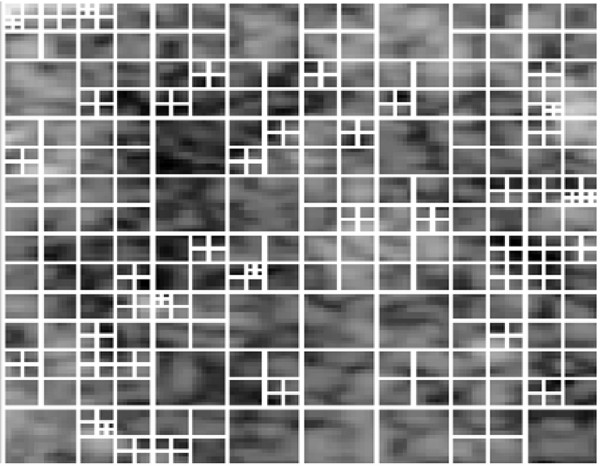
**Result of square-tree decomposition performed on*L***_***S***_**from Figure**6, where*w*(7) = 0*1/(*M*_s_*N_***s***_),*w*(8) = 32*2/(*M*_s_*N_***s***_),*w*(9) = 152*4/(*M*_s_*N_***s***_),*w*(10) = 148*8/(*M*_s_*N_***s***_**) were obtained.** Smaller squares exist in places of big brightness changes. Larger squares appropriately in places, where the image brightness does not change by more than the set threshold. The image is therefore divided into square areas within which the difference between the minimum and maximum values is below the set threshold. If the difference between these values (minimum and maximum) is greater than the threshold, then the area is further divided. The division is always made into four smaller (square) areas. This procedure is carried out until the area reaches the size of 1×1 and a further division is not possible, or if the criterion of permissible differences in brightness is met.

The square-tree decomposition is usually associated with image compression [25]. However, due to its properties, it can also be used to determine global features of images [25]. The name of this decomposition comes from the specificity of its operation. Namely, the analyzed area is divided into smaller and smaller squares until at certain criterion related to the values of pixels in the analyzed square is met. As a result of such a course of action, a tree subdivision of the right size at each level is created. The relevant picture of the thyroid *L*_*S*_ with the resolution *M*_*s*_×*N*_*s*_ can be divided into “*i*” rectangular areas *L*_*i*_ with the resolution *M*_*i*_×*N*_*i*_ for the "*i*" coefficient value in the range 1 < = *i* < = *I*. Thus, the smallest area may have a pixel size, that is 1×1, and the largest - *M*_*s*_×*N*_*s*_ pixels. In practice, the resolution of the image *L*_*S*_ is adjusted to a resolution which is the power of 2. Then, assuming a suitable threshold, it is divided into areas such as 8×8, 4×4, 2×2 and 1×1 in accordance with the rules described. An example of a division is shown in the figure below (Figure 6, Figure 7)

Individual values of the features *w*(7) to *w*(10) are calculated as a percentage share against the whole image. For example, the image *Ls* shown in Figure 7 contains 0 areas of size 1×1 (*w*(7) = 0), 32 areas of size 2×2 ( *w*(8) = 32*2/( *M*_s_*N_*s*_)), 152 areas of size 4×4 (*w*(9) = 152*4/( *M*_s_*N_*s*_)) and 148 areas of size 8×8 (*w*(10) = 148*8/( *M*_s_*N_*s*_)). The number of areas and, thus, the value of features *w*(7), *w*(8), *w*(9) and *w*(10) is highly dependent on the number of instances of objects (bright or dark) and their size. Large homogeneous objects enhance the value of the features *w*(10), *w*(9), medium-sized objects - *w*(8), and small objects - *w*(7). It should be noted here that the division into the smallest areas (size 1×1) also occur on contrasting edges. Thus, their number is not always strictly proportional to the number of small objects on the thyroid lobe.

#### Justification for the selection of features *w*(1) to *w*(10)

All 10 features were selected following medical conditions. The analyzed ROI areas (images *Ls*) contain numerous artifacts which have a high impact on the outcome. The features *w*(1) to *w*(10) were chosen in such a way that elimination of artifacts provide a new image feature. The artifacts associated with both small bright nodules and other bright inclusions were removed in *w*(2). If the structure of the image *Ls* is non-uniform, there are no strict medical reasons for its analysis. For this reason, the features *w*(7) to *w*(10), which divide the image *Ls* into areas *L*_*i*_, were suggested. The number of these areas (of particular sizes) indicates additional features of the image. The 10% threshold was chosen based on the difference in the minimum and maximum values of the analyzed group of images. For the threshold value of 20, 30 or 40% (or more), almost zero values of the features *w*(7) to *w*(10) were obtained. For values below the threshold of 10%, oversegmentation was obtained. To measure the homogeneity from the global, other tools such as GLCM and FFT were used. The features *w*(5) and *w*(6) determine the location and number of pixels in the area *L*_*GLCMB*_. They are, thus, a measure of the spread of contrast in an image (feature *w*(6)) and information about its center of gravity (feature *w*(5)). These features are necessary when the amount of colloid in follicles decreases and when the number of cellular elements increases. Then, the image *Ls* is characterized by varying degrees of contrast which is less capable of being detected by the features *w*(7) to *w*(10). The noise in the image caused by small artifacts does not exceed 10%. For this reason, the binarization threshold chosen for the matrix GLCM was 0.1. In extreme cases, the image *Ls* may have follicles with very mild edges. Then, *w*(1) is a reliable feature because the features *w*(5) to *w*(6) *w*ill not be effective here. The feature *w*(3) associated with smoothness and uniformity of the image *Ls* is comparable in its properties. Because of the afore-mentioned artifacts (usually in the form of clear follicles), a direct measurement of the average brightness of pixels in an image *Ls* may not produce satisfactory results. For this reason, the feature *w*(4) was defined as the one which treats 10% of the darkest pixels as noise and, thus, they are not taken into account. Depending on the form of a texture (of the image *Ls*), the defined features favour a selection of its features. The values of the features *w*(1) to *w*(10) are also, in varying degrees, sensitive to the image *Ls* specifity. They favour a low contrast, remove noise, are a measure of brightness, cyclicality in the image or its smoothness.

#### Initial verification of features

**Figure 8:**
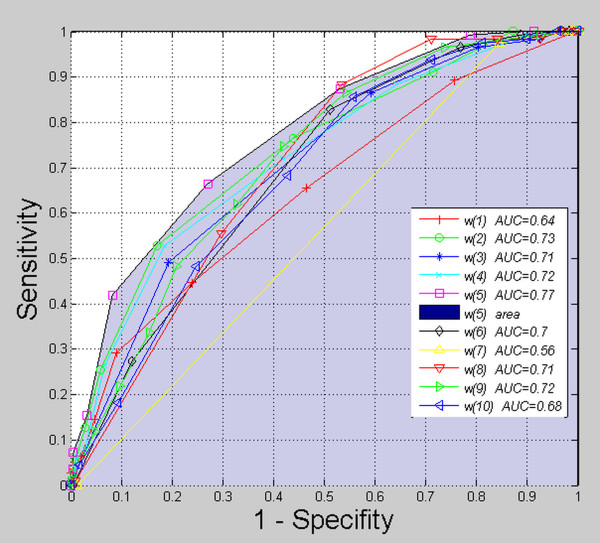
***ROC*graph (Receiver Operating Characteristics).** The graph was obtained for all 376 images (29 healthy subjects and 65 patients with Hashimoto’s disease). The analysis concerns the impact on the result of each of the features *w*(1) to *w*(10) separately. The graph shows that the feature *w*(5), appearing alone as a criterion for the division of healthy and ill subjects, gives the best results. For this feature, the area under the *ROC* curve is 0.77. This graph only shows the impact of each feature ( *w*(1) to *w*(10)) on the efficiency of separation of healthy subjects from patients. It can be easily noticed that the obtained results are not very good. It will be proved further on that these results get better to a considerable extent when several features are taken into account simultaneously and the decision tree is created. Selection of the most representative features for the construction of the decision tree is the subject of further consideration.

All of the features *w*(1) to *w*(10) are the basis for building an expert system. Initially, however, a verification of influence of each of the features from *w*(1) to *w*(10) was made. It is worth emphasizing that each one of them was considered separately. For this purpose, the measurements of *ROC* curves (Receiver Operating Characteristics) were carried out for all 376 images (29 healthy subjects and 65 patients with Hashimoto’s disease). *ROC* function was calculated as the dependence of sensitivity on specifity. Assuming *FN* as false negative cases, *FP* as false positive, *TP* as true positive and *TN* as true negative, sensitivity was calculated as *TP*/( *TP* +  *FN*) and specifity as *TN*/( *FP* + TN). The obtained results are shown in Figure 8. Each of the measurement points was obtained by changing the value of individual features from the minimum to maximum value in increments of 10%. The area under the *ROC* curve - *AUC* (Area Under Curve), is also marked in the same figure. *AUC* values lie within the range of 0.56 to 0.77 for each of the features *w*(1) to *w*(10) measured separately. The maximum value ( *AUC* = 0.77) was marked with a darkened area and occurs for the feature *w*(5). A separate analysis of each feature *w*(1) to *w*(10) does not give a true picture of their impact on the result. Therefore, the features *w*(1) to *w*(10) will be considered together later in this paper. Their impact on the final result will also be further examined in a broader context.

## Results

**Figure 9:**
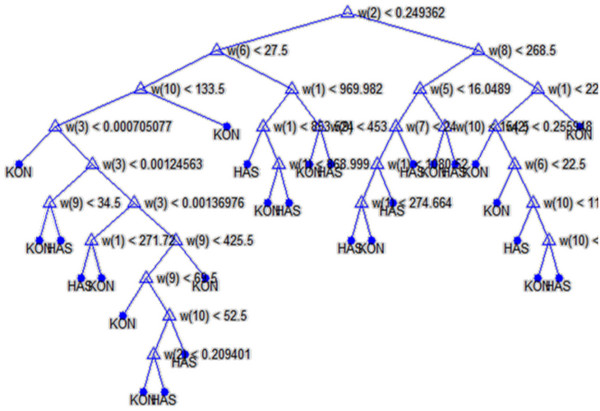
**Full decision tree.** The resultant decision tree formed for all features. „KON” and „HAS” values stand for the classification result – healthy, sick person, respectively. This is one of many decision trees created during the analysis of “ *w*” features significance level. The feature *w*(2) related to the regional minimum value in the *Ls* image is in the first node of the decision tree. Next, in the subsequent nodes, there are the features *w*(6) and *w*(8) associated with the neighbourhood matrix *L*_*GLCM*_ and the square-tree decomposition of the image *Ls*. Subsequent nodes are associated with the other features *w*(1), *w*(3), *w*(4), *w*(5), *w*(7), *w*(9) and *w*(10).

Because of the functionality, the IT facilities prepared and a possibility of easy visualisation, the classifier in the form of decision trees has been chosen. In all cases a non-parametrical algorithm creating CART (Classification and Regression Trees) binary trees has been used as the method for decision trees induction. An increase in the nodes purity has been used as the criterion assessing the quality of CART divisions. The Gini index has been used as the measure of nodes impurity. Because of a small number of cases the tree creation was not limited by a minimum number of vectors in a node. As the considerations apply to the construction of a classifier for patients and healthy persons based on the knowledge base – *w*(1) to *w*(10) features, a preliminary prepared tree Figure 9 was built based on the full information, using the training group.

**Figure 10:**
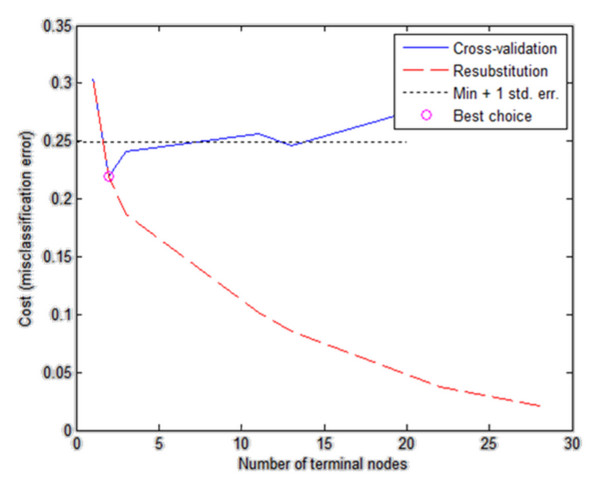
**Graph of classification error changes versus the number of nodes, taking into account the place, where the decision tree was trimmed (best choice).** Two cross-validation curves have been shown – the blue colour. The resubstitution is marked red. In addition, the cut off line and the best selection point (red circle) have been marked. The presented graph shows that best choice occurs for cost (misclassification error) equal to 2.15 when the number of terminal nodes is 2. This choice is correct because it is the minimum error for cross validation.

Then, based on the results obtained in the validation group (Figure 10), the tree was pruned. The best tree is the one that has a residual variance, that is no more than one standard error above the minimum value along the cross-validation line (Figure 10). The obtained results are shown in the table below (Table 1).

**Table 1:** Specification of results obtained for a typical and a trimmed decision tree

**Tree**	***TP*- true positive**	***FN*- false negative**	***TN*- true negative**	***FP*- false positive**
Typical	127	3	56	2
Cut	108	22	39	19

**Figure 11:**
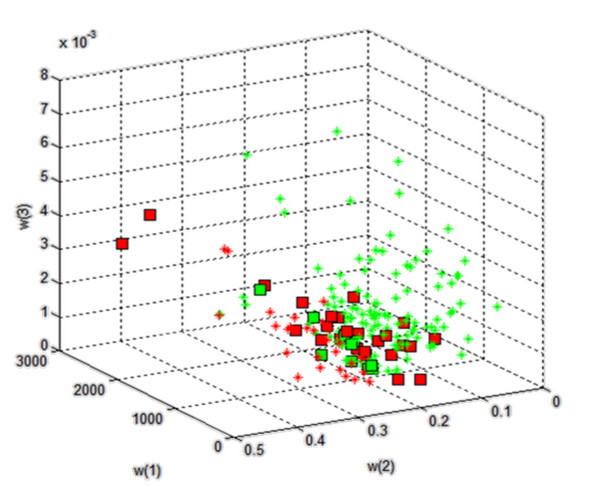
**The result of classification using a decision tree created for 10 features**. Classification errors are marked using squares. For this rotation of coordinate system the issues related to the common part of both separated groups are roughly visible. When selecting other “ *w*” features in the visualisation, this boundary is even less visible. Looking at these examples of the features *w*(1), *w*(2) and *w*(3) on each axis, a possible separation of healthy subjects from patients is visible. For other features, as shown further on, these results are much better. For a full visualisation of all the features, a 10-dimensional space should be used.

Table 1 contains the results obtained from the decision tree which was created on the basis of different features (*w*(1) to *w*(10)). The classification results are also shown in a 3D diagram in Figure 11 where only three out of ten features, i.e. *w*(1), *w*(2) and *w*(3), are on the axes. Classification errors are indicated on the graph with square points.

**Figure 12:**
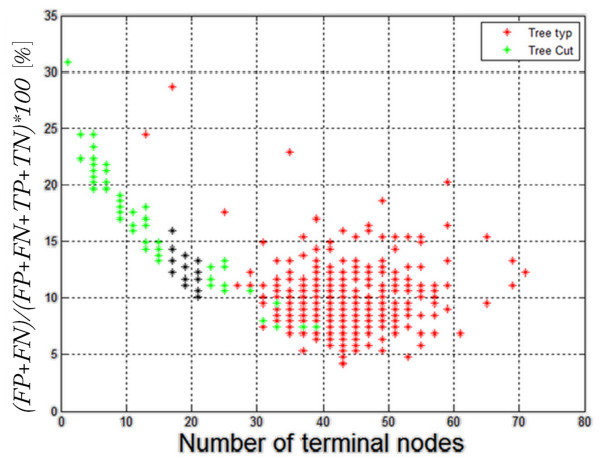
**Dependence of classification error (the ratio of erroneously classified to all examples) on the number of decision tree nodes. Red – not trimmed trees, green – trimmed decision trees and black – optimal trees.** The selection of optimum trees consists in the minimisation of the terminal nodes number at parallel minimisation of the classification error. The optimum trees are those, which are marked black and their terminal nodes number ranges between 16 and 22. Optimum has been chosen in the place where the error is not reduced and the number of terminal nodes is the smallest. A shift in any direction affects the inflated number of terminal nodes, or substantially increases the error. The results marked in red show that non-trimmed decision trees over-fit to the data reaching a smaller error at the expense of the number of terminal nodes.

The representative attributes *w*(1) to *w*(10) were chosen by creating 2^10^ decision trees. To do it, all possible configurations of features *w*(1) to *w*(10) were used. Then, based on the results obtained in the validation group, the tree was cut. As a result, 2*2^10^ = 2048 decision trees were obtained. Table 2 shows the ones for which the slightest error in the test group and the smallest number of nodes were obtained (black dots on the graph in Figure 12). The following symbols were adopted: *TP*- true positive, *TN*- true negative, *FP*- false positive, *FN*- false negative, *w* – feature: “0” does not exist, “1” exists in the creation of a tree. Adopting such designations ("0" or "1"), the attributes occurring in the creation of a tree can be, in short, written as one number (No) within the range 1 to 1023. The number was formed from a binary record of the occurrence or non occurrence of attributes. For example, the value No = 64 means that only one attribute, that is *w*(7), occurred in the tree creation. The results presented in Table 2 are the only basis for the selection of the final form of the tree. On the other hand, Figure 12 shows the dependence of classification error (the ratio of misclassified examples to all examples) as a function of the number of nodes of a decision tree.

**Table 2:** **Fragment of the best results obtained for “*w*” features (marked with black dots on the graph - Figure**12**), for a typical and trimmed classification tree and classification errors*TP*,*FN*,*TN*,*FP*with the number of tree nodes**

	***w***										**Tree typical**							**Tree cut**						
**No**	**1**	**2**	**3**	**4**	**5**	**6**	**7**	**8**	**9**	**10**	***TP***	***FN***	***TN***	***FP***	**node**	***ACC*[%]**	***FN*** **+** ***FP*[%]**	***TP***	***FN***	***TN***	***FP***	**node**	***ACC*[%]**	***FN*** **+** ***FP*[%]**
143	1	1	1	1	0	0	0	1	0	0	124	6	52	6	41	94	6.38	123	7	46	12	21	90	10.11
822	0	1	1	0	1	1	0	0	1	1	125	5	55	3	43	96	4.26	123	7	46	12	21	90	10.11
647	1	1	1	0	0	0	0	1	0	1	120	10	54	4	39	93	7.45	124	6	44	14	21	89	10.64
759	1	1	1	0	1	1	1	1	0	1	125	5	51	7	41	94	6.38	125	5	43	15	21	89	10.64
786	0	1	0	0	1	0	0	0	1	1	127	3	49	9	45	94	6.38	124	6	44	14	21	89	10.64
912	0	0	0	0	1	0	0	1	1	1	126	4	51	7	47	94	5.85	127	3	40	18	19	89	11.17
984	0	0	0	1	1	0	1	1	1	1	127	3	48	10	43	93	6.91	124	6	43	15	19	89	11.17
620	0	0	1	1	0	1	1	0	0	1	127	3	46	12	33	92	7.98	124	6	42	16	21	88	11.7
941	1	0	1	1	0	1	0	1	1	1	127	3	50	8	43	94	5.85	122	8	44	14	21	88	11.7
1010	0	1	0	0	1	1	1	1	1	1	124	6	49	9	37	92	7.98	123	7	43	15	19	88	11.7
135	1	1	1	0	0	0	0	1	0	0	124	6	52	6	41	94	6.38	123	7	42	16	17	88	12.23
167	1	1	1	0	0	1	0	1	0	0	115	15	56	2	33	91	9.04	123	7	42	16	17	88	12.23
710	0	1	1	0	0	0	1	1	0	1	123	7	50	8	37	92	7.98	127	3	38	20	17	88	12.23
873	1	0	0	1	0	1	1	0	1	1	127	3	47	11	41	93	7.45	124	6	41	17	21	88	12.23

In the case of a pruned tree, the first lines 1 through 5 in Table 2 indicate an error within the range of 10.11 to 10.64%. In this range, the number of attributes – starting from the first line - is 5, 6, 5, 8, 4. Taking into account the minimization of the features involved in the creation of a classifier (and, thus, the speed of computation), and a classification error of 10.64%, by only 0.54% larger than the smallest error obtained - this decision tree was identified as the final one. For the pruned decision tree, selected in this way, the following results were obtained: a classification error *FN* +  *FP* at 10.64%, *TP* = 124, *FN* = 6, *TN* = 44, *FP* = 14, 21 nodes and features *w*(2), *w*(5), *w*(9) and *w*(10). For this decision tree, the following results for each indicator were obtained: sensitivity or True Positive Rate *TPR* =  *TP*/( *TP* +  *FN*) = 0.95, False positive Rate *FPR* =  *FP*/( *FP* +  *TN*) = 0.241, *ACC*uracy *ACC* = ( *TP* +  *TN*)/( *TP* +  *TN* +  *FP* +  *FN*) = 0.89, specificity or True Negative Rate *TNR* =  *TN*/( *FP* +  *TN*) = 0.75, Positive Predictive Value *PPV* =  *TP*/( *TP* +  *FP*) = 0.89, Negative Predictive Value *NPV* =  *TN*/( *TN* +  *FN*) = 0.88, False Discovery Rate *FDR* =  *FP*/( *FP* +  *TP*) = 0.1.

When analyzing the optimal set of features existing in this decision tree (*w*(2), *w*(5), *w*(9) and *w*(10)), it can be seen that these are the features associated with minimum brightness ( *w*(2) and *w*(5)) and texture features such as areas (to be more exact, their percentage number). In this case, the areas are 4×4- *w*(9) and 8×8- *w*(10). Therefore, general conditions for the indication of representative features related to the analysis and image processing can be given. These are two features: the brightness value *w*(5) with the minimum value *w*(2) and the number of homogeneous areas 4×4 and 8×8 for the assumed brightness threshold of 10%.

While the representativeness of dark areas of the thyroid lobe has been repeatedly demonstrated in numerous papers [6,8,10,12,21,26-28], diagnostic reliability of homogeneity (features *w*(9), *w*(10)) still requires a comment. The number of homogeneous areas 4×4 and 8×8 is strongly dependent on the brightness threshold. In this case, it was a 10% threshold adopted arbitrarily. As the threshold limit determines the boundary between the minimum and maximum of all the pixels present in the shared area, the division is not dependent on the absolute level of brightness. On the other hand, the number of areas 1×1, 2×2, 4×4, 8×8, 16×16 is closely dependent on the adopted threshold and so, inter alia, the value of the features *w*(9) and *w*(10). By increasing the threshold, the number of large areas increases (8×8, 16×16) and, the other way round, by reducing the threshold, the number of small areas (1×1, 2×2) decreases. In each case, the obtained results and their representativeness is relative to the size of thyroid follicles. For this reason, a threshold of 10% was adopted.

From the diagnostic point of view, the results obtained by using only one feature are also interesting. These results are shown in Table 3.

**Table 3:** Results obtained for one feature out of ten in the tree creation

	***w***										**Tree typical**											
**No**	**1**	**2**	**3**	**4**	**5**	**6**	**7**	**8**	**9**	**10**	***TP***	***FN***	***TN***	***FP***	**node**	***FDR***	***TPR***	***FPR***	***ACC***	***TNR***	***PPV***	***NPV***
1	1	0	0	0	0	0	0	0	0	0	119	11	44	14	69	0.105	0.915	0.241	0.867	0.758	0.89	0.800
2	0	1	0	0	0	0	0	0	0	0	125	5	44	14	55	0.100	0.961	0.241	0.898	0.758	0.899	0.898
4	0	0	1	0	0	0	0	0	0	0	123	7	44	14	69	0.102	0.946	0.241	0.88	0.758	0.897	0.862
8	0	0	0	1	0	0	0	0	0	0	119	11	23	35	13	0.227	0.915	0.603	0.755	0.396	0.772	0.676
16	0	0	0	0	1	0	0	0	0	0	119	11	48	10	59	0.077	0.915	0.172	0.888	0.827	0.922	0.813
32	0	0	0	0	0	1	0	0	0	0	117	13	28	30	35	0.204	0.900	0.517	0.771	0.482	0.795	0.682
64	0	0	0	0	0	0	1	0	0	0	120	10	14	44	17	0.268	0.923	0.758	0.712	0.241	0.731	0.583
128	0	0	0	0	0	0	0	1	0	0	115	15	44	14	65	0.108	0.884	0.241	0.845	0.758	0.891	0.745
256	0	0	0	0	0	0	0	0	1	0	121	9	44	14	71	0.103	0.930	0.241	0.877	0.758	0.896	0.830
512	0	0	0	0	0	0	0	0	0	1	122	8	28	30	59	0.197	0.938	0.517	0.797	0.482	0.802	0.777

From the obtained results (Table 3), the order of features for which the smallest *FN* error was obtained is clearly seen, i.e. *w*(2), *w*(3), *w*(10), *w*(9) etc. This means that the feature *w*(2) is the most representative. Of course, using only the feature *w*(2) in the classification introduces a significant error when, for example, compared to using some other features (as shown, for example, in Table 2 for the number 786). For this reason, Table 3 should be considered for reference only and it ends the discussion and results concerning the classification.

### Assessment of features significance

**Figure 13:**
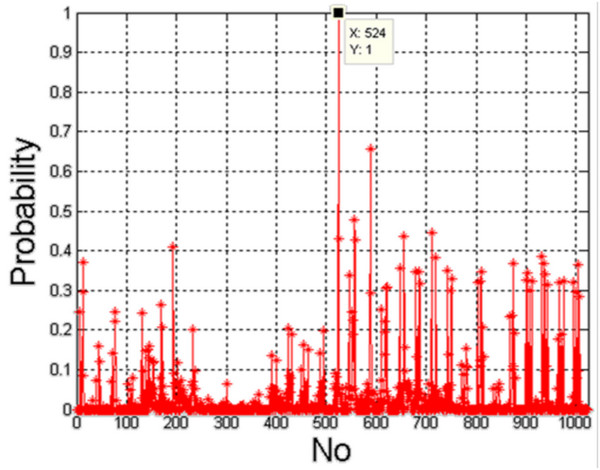
**The graph of occurrence frequency (probability) for individual feature configurations (1 to 1023).** The graph shows clearly the most frequent occurrence of features combination for tree number 524. The other decision trees, formed in other feature configurations, occur less frequently. A specific configuration (1 to 1023) is a particular configuration of the features involved in the formation of a decision tree. These values can easily be read taking into account the fact that each of the features *w*(1) to *w*(10) may define the next bit of a 10-bit recording. For example, 16 is only the occurrence of the feature *w*(5), 32 - *w*(6) and 524 - *w*(3), *w*(4) and *w*(10). The graph shows that the highest incidence was observed for the features *w*(3), *w*(4) and (10) together.

All the results presented so far concern the creation of 2*2^10^ decision trees. As mentioned above, each tree was created for various configurations of features *w*(1) to *w*(10). During the creation of each tree, the division into a learning, validation and testing vectors was conducted at random. Thus, the question of impact of randomly selected cases on the results becomes important. For this purpose, the creation of 2*2^10^ decision trees was repeated 2000 times, which gave more than 4 million decision trees. During this process, the frequency of features *w*(1) to *w*(10) in the selection of the optimal tree and the frequency of occurrence of any of their 1023 configurations were checked. The results are shown below (Figure 13, Table 4).

**Table 4:** **Percentage frequency of features*w*(1) to*w*(10) occurrence in the optimum tree (the sum gives 100%)**

***w*(1)**	***w*(2)**	***w*(3)**	***w*(4)**	***w*(5)**	***w*(6)**	***w*(7)**	***w*(8)**	***w*(9)**	***w*(10)**
13%	11%	11%	12%	2%	10%	8%	11%	7%	14%

Figure 13 shows the frequency of a particular configuration of features - values in the range 1 to 1023. The graph indicates that configuration 524 is most common. It can be easily checked via binary calculations that these are the features which take part in the creation of a decision tree, namely *w*(3), *w*(4) and *w* (10). What is more, the summary of the frequency of individual features gives complete information on their relevance. The analysis of the table presented in Table 4 shows that the most important feature is *w*(10) having a 14% share in comparison to other features. Together with the information presented on the graph in Figure 13 (showing the occurrence of features *w*(3), *w*(4) and *w*(10)), it can be easily calculated that these features have a cumulative of 11 +12 +14 = 37% correct results. This means that they are also the most representative in the assessment of Hashimoto's disease. The features *w*(3), *w*(4) and *w*(10) are determined by smoothness, the minimum brightness and the number of areas 8×8, isolated in the process of square-tree decomposition. They are exactly the same as the characteristic parameters for Hashimoto’s disease described in chapter above.

From a practical point of view, the configurations of features on the basis of which an optimal tree has never been created are also interesting. These are all values (configurations of features) shown in Figure 13 for which the probability is zero (Table 5).

**Table 5:** **Percentage frequency of features*w*(1) to*w*(10) occurrence in the case of decision trees, which did not meet the optimality criterion (the sum gives 100%)**

***w*(1)**	***w*(2)**	***w*(3)**	***w*(4)**	***w*(5)**	***w*(6)**	***w*(7)**	***w*(8)**	***w*(9)**	***w*(10)**
8.7%	9.6%	9.9%	9.7%	11.8%	10.7%	10.8%	6.8%	12.2%	9.4%

The results presented in the table in Table 5 show that there is no feature with a significant and distinctive frequency of occurrence. This means that a specific configuration of features decided about the absence of a decision tree. For this reason, pairs of features for all configurations were analyzed. Moreover, the percentage of all the trees which do not meet the condition of optimality was calculated (Table 6). The results show that the feature *w*(9) in tandem with one of the features *w*(1), *w*(2), *w*(3), *w*(4), *w*(5), *w*(6) or *w*(7) has the largest share. Together with the information given in Table 5 and Table 6, it must be clearly stated that the feature *w*(9) is irrelevant from the diagnostic point of view. At the same time, the selection of the feature *w*(10) as the most important is confirmed. This feature together with *w*(3) and *w*(4) gives satisfactory results, which are presented above.

**Table 6:** Percentage frequency of feature pairs occurrence as compared with all not qualified trees (not meeting the optimality requirements)

**Pairs**	**Percentage frequency**
w(6) andw(9)	81%
*w*(5) and *w*(9)	81%
*w*(7) and *w*(9)	81%
*w*(3) and *w*(9)	80%
*w*(4) and *w*(9)	79%
*w*(5) and *w*(7)	79%
*w*(2) and *w*(9)	78%
*w*(6) and *w*(7)	77%
*w*(1) and *w*(9)	77%
*w*(3) and *w*(5)	77%
*w*(5) and *w*(6)	77%

### Comparison with other authors results

Papers of other authors can be divided into two groups. In the first one, there are results profiled to the analysis of Hashimoto's disease. In these papers, texture analysis does not appear or it is covered, but to a lesser extent. In the latter one, there are papers dominated by texture analysis but it is not profiled for the diagnosis of Hashimoto's disease. In these papers, the results of analysis are strongly dependent on the chosen methodology of procedure and the suggested approach. For example, nodule detection described in paper [29] achieved pixelwise classification accuracy of 74.2%. Similarly, in paper [30], the best nodule detection accuracy, obtained with the SVM classifier, was 87.6%. In more recent papers, Smutek et al. show sensitivity and specificity of 100% (paper [14]). The same author in paper [31] profiles texture analysis to Hashimoto’s disease to obtain *ACC* equal to 92%. However, the analyzed area is not rectangular and is not marked fully automatically. In earlier works, Cavouras et al. [32] report 93.7% classification accuracy for distinguishing normal and abnormal livers. Likewise, Horng et al. [33] report 83.3% classification accuracy. In each case, the authors apply texture analysis to the area marked manually. This area is usually rectangular, which is not practical. The results profiled to Hashimoto’s disease are present in only a few papers [2,7-9,11] and they show *ACC* of 90%. They are worse than the results obtained in this work ( *ACC* = 94% for a full decision tree and *ACC* = 89% for a pruned decision tree - Table 2). Additionally, in this paper, new configurations of features are taken into account and their impact on classification results are presented.

Our results were also compared with the ones in paper [9]. G. Mazziotti writes that he obtained *ACC* equal to 87%. This measurement was carried out in paper [9] using images obtained in gray levels and analyzed in Corel PHOTO-PAINT. Using only the average brightness of the selected area in the thyroid image as a feature, the obtained results were inferior to the results published in paper [9] i.e.: *ACC* = 75%. It was mainly caused by taking into account a different scope of ROI area in the thyroid image. In comparison with, for example, the feature *w*(4), the result of *ACC* is 72% (Figure 8). The situation changes when comparing the results obtained from the methods of classification. In this case, the results obtained in this paper are much better (*ACC* = 94%) owing to the features *w*(1) to *w*(10). Thus, in the assessement of Hashimoto’s disease, more than one feature should be taken into account. Image analysis should be performed directly on DICOM file in the profiled software.

## Summary

In the paper, 10 features derived for texture images of the thyroid lobe were analyzed. This analysis resulted in the following conclusions:

· reducing the number of measured features from 10 to 3 enables to obtain a classification error of 11%,

· on the basis of the analysis of diagnostic significance of features, it was shown that the most important feature is *w*(10) and the least important one is *w*(9),

· additional conditions for an ultrasound specialist related to the image analysis of thyroid lobes were identified (significance of features),

· analysis time per patient was optimized to tens of milliseconds (for Intel Core 2 Quad CPU Q9300@2.5 GHz),

· on the basis of analysis of the features, it was clearly demonstrated that the value of the minimum brightness of the thyroid lobe image (after the removal of noise) and the number of artifacts (nodules, etc.) are most representative in the diagnosis of Hashimoto's disease,

· a detailed analysis of the features showed that the feature *w*(9) is irrelevant from the standpoint of diagnosis whereas the feature *w*(10) is most important and, in conjunction with the features *w*(3) and *w*(4), gives satisfactory results.

## Competing interests

The authors declare that they have no competing interests.

## Authors’ contributions

RK and ZW suggested the algorithm for images analysing and processing, implemented it and analysed the images USG. WZ concept of image analysis in Hashimoto, the collection and analysis of literature, performance studies and correlate the images with the results of biochemical, participation in the analysis. JM performance of the study, participation in the collection of literature, the supervision of the base at the time of collecting material. BS performance of the study, preparation of material for analysis. WW coordination of the whole team, consultation to create the project. All authors have read and approved the final manuscript.
